# *Emv2*, the only endogenous ecotropic murine leukemia virus of C57BL/6J mice

**DOI:** 10.1186/1742-4690-9-23

**Published:** 2012-03-22

**Authors:** George R Young, George Kassiotis, Jonathan P Stoye

**Affiliations:** 1 Division of Immunoregulation, MRC National Institute for Medical Research, London, UK; 2 Division of Virology, MRC National Institute for Medical Research, The Ridgeway, Mill Hill, London NW7 1AA, UK

## Abstract

With the proliferation of sequence data, great challenges are posed in the correct annotation of endogenous retroviruses, which together comprise up to ten per cent of the genomes of many organisms. It is therefore essential that all sources of information are carefully considered before drawing conclusions concerning the phylogeny, distribution and biological properties of endogenous retroviruses. We suggest that such due diligence has not been applied in the description of an endogenous ecotropic retrovirus that recently appeared in *Retrovirology*.

## Commentary

We are writing in regard to the paper by Lee *et al. *[1], recently published in *Retrovirology*. We have a number of concerns about the accuracy of this paper that need to be considered.

First we would like to ask whether the virus identified is novel or of intact coding potential. In the pioneering study by Jenkins *et al. *[2], they examined the endogenous ecotropic proviruses (*Emv *loci) present in the germ line of inbred mice. Different inherited proviruses were defined by Southern hybridization analysis using an ecotropic murine leukemia virus-specific envelope gene probe (pEco) and DNA from different mouse strains cut with several different restriction enzymes to yield a variety of virus-flanking sequence fragments. Different proviruses, integrated at different chromosomal locations, will yield characteristic sets of fragments [2]. *Emv2 *was found in C57BL/6J and related strains. It was characterized by pEco reactive fragments of 5.2, 7.0, 11.5, 17.0 kb in DNA digested with *Pvu*II, *Hind*III, *Xba*I and *Bcl*I respectively [2,3]. A number of genetic studies show that *Emv2 *maps to the distal region of mouse chromosome 8 http://www.informatics.jax.org/searches/mapdata_report_by_marker.cgi?8452, and whilst not yet annotated on the C57BL/6J reference genome assembly, its location can be ascertained by BLASTn searches of the genomic sequence with the pEco probe sequence. This reveals only a single match in this region of chromosome 8 for the entire C57BL/6J genome. Although a fraction of old C57BL/6 mice express ecotropic virus [4] and a poorly inducible ecotropic virus has been mapped to the distal region of chromosome 8 of both C57BL6 [5] and C57BL/10 [6] mice, transfection studies clearly indicate that the *Emv2 *provirus is replication defective due to a specific base change in *pol *that results in an Ala to Pro change in RT [3,7,8]. Presumably correction of this defect is readily and routinely accomplished, perhaps by recombination with another endogenous virus [9].

In the paper by Lee *et al. *[1], an ecotropic provirus also mapping to chromosome 8 was found by mining the v37 build of the C67BL/6J genome sequence. They refer to this provirus as novel, calling it 'ERV_mch8_', and pointing to the absence of relevant annotation and sequence information in the NCBI databases to establish its relationship to *Emv2*. However, we note that the sequence predicts that 'ERV_mch8_' would give rise to pEco reactive fragments of 4.9, 7.1. 11.2 and 15.1 kb following digestion with *Pvu*II, *Hind*III, *Xba*I and *Bcl*I, it carries the G to C change resulting in the inactivating pol mutation and maps to the same region of mouse chromosome 8 that carries the only ecotropic murine leukemia provirus present in B6 mice. Thus, there seems no reason to invoke a novel ecotropic provirus. Further, to label this virus as possessing "intact coding potential" also seems misleading given its defective nature.

We would further like to point out the need for great care in classifying murine leukemia viruses for phylogenetic purposes. In Figure one of the paper, a tree is shown with "polytropic" (U13766) and "xenotropic" (DQ399707) MLVs. Indeed, although these sequences are likely to encode viruses that are polytropic and xenotropic in host range, neither is endogenous. Instead, inspection of the NCBI annotations reveals that both are recombinants (U13766 is a class I MCF virus and thus likely to contain endogenous ecotropic, polytropic and xenotropic sequences see [10] and DQ399707 is XMRV clone VP62, most probably a polytropic/xenotropic recombinant [11]). Better choices for such an analysis would be found among the endogenous non-ecotropic ERV sequences listed in [12]. This problem is also seen in Figure two. It shows a comparison between four *gag *sequences, two of which are endogenous (*Emv1 *and *Emv2*), and two that should not be thought of as endogenous but are replication competent viruses most probably derived by recombination between *Emv1 *(Nerv) or *Emv2 *(MelARV) and one or more non-ecotropic viruses [5,9]. Recombination has converted these viruses to B-tropism, allowing replication in A/J or B6 derived cells or tissues, and may also be responsible for repairing the *pol *mutation.

Jenkins *et al. *defined the strain distribution of different endogenous ecotropic proviruses, including *Emv2*, by Southern hybridization [2]. They found *Emv2 *only in C57BL/6J and a few closely related strains (C57BL/KsJ, C57BL/6ByJ, C57BL/10 J, C57BL/l0SnJ, C57BR/cdJ). By contrast, Lee *et al.*, using a PCR based approach and primers that are wrongly presumed specific for the 'ERV_mch8_' sequence, found an ecotropic provirus on chromosome 8 in a wide variety of strains. Unfortunately, the primers selected both lie within the provirus itself, and sequence comparisons predict that they are unable to distinguish between *Emv2 *and at least two other Emvs (*Emv1 *and *Emv11*). Unambiguous identification of specific proviruses by PCR is most easily accomplished using one primer designed to the provirus and one corresponding to sequences flanking its integration site. Use of two internal viral primers is likely to give a positive signal with mouse DNAs containing any Emv proviruses. This fact makes the data presented in Figure three of Lee *et al. *regarding the distribution of endogenous ecotropic proviruses as well as any discussion based on them wholly unreliable. For example, their data indicate a shared ecotropic provirus in C3H/HeJ and C57BL/6J mice; this is inconsistent with the molecular demonstration of two independently segregating proviruses in BXH recombinant inbred mice [13]. Parenthetically, we also note that the use of the same primers in expression studies provides no control against the presence of DNA contamination or amplification of unrelated transcripts; it would have been highly desirable to use primers spanning the viral intron or starting material from a strain containing another *Emv *as controls.

We consider that these shortcomings should have been obvious from a consideration of the appropriate literature, and we caution against the blind acceptance of genome annotations. One might have hoped these weaknesses would have been identified before publication.

## Competing interests

The authors declare that they have no competing interests.

## Authors' contributions

GRY, GK and JPS contributed equally to the preparation of this letter. All authors read and approved the final manuscript.
